# Payday lending in the UK: the regul(aris)ation of a necessary evil?

**DOI:** 10.1017/S0047279416000015

**Published:** 2016-07

**Authors:** KAREN ROWLINGSON, LINDSEY APPLEYARD, JODI GARDNER

**Affiliations:** * School of Social Policy, University of Birmingham, Edgbaston, Birmingham, B15 2TT email: K.Rowlingson@bham.ac.uk; ** Centre for Business in Society, Coventry University, Priory Street, Coventry, CV1 5FB email: ac1113@coventry.ac.uk; *** Corpus Christi College, Merton Street, Oxford, OX1 4JF email: jodi.gardner@ccc.ox.ac.uk

## Abstract

Concern about the increasing use of payday lending led the UK's Financial Conduct Authority to introduce landmark reforms in 2014/15. While these reforms have generally been welcomed as a way of curbing ‘extortionate’ and ‘predatory’ lending, this paper presents a more nuanced picture based on a theoretically-informed analysis of the growth and nature of payday lending combined with original and rigorous qualitative interviews with customers. We argue that payday lending has grown as a result of three major and inter-related trends: growing income insecurity for people both in and out of work; cuts in state welfare provision; and increasing financialisation. Recent reforms of payday lending do nothing to tackle these root causes. Our research also makes a major contribution to debates about the ‘everyday life’ of financialisation by focusing on the ‘lived experience’ of borrowers. We show that, contrary to the rather simplistic picture presented by the media and many campaigners, various aspects of payday lending are actually welcomed by customers, given the situations they are in. Tighter regulation may therefore have negative consequences for some. More generally, we argue that the regul(aris)ation of payday lending reinforces the shift in the role of the state from provider/redistributor to regulator/enabler.

## The regul(aris)ation of payday lending in the UK

Payday lending increased dramatically in the UK from 2006–12, causing much media and public concern about the extremely high cost of this particular form of short-term credit. The original aim of payday lending was to lend a small amount to someone in advance of their payday. Once they received their wages, the loan would be repaid. Such loans would therefore be relatively small amounts over a short time period. Other forms of high-cost, short-term credit (HCSTC) include doorstep/weekly collected credit and pawnbroking but these have not received the same level of public attention as payday lending in recent times. This paper therefore focuses particularly on payday lending which, despite all the public attention, has received remarkably little attention from social policy academics in the UK.

In a previous issue of the *Journal of Social Policy*, Marston and Shevellar (2014: 169) argued that ‘the discipline of social policy needs to take a more active interest in . . . the underlying drivers behind this growth [in payday lending] and [the] implications for welfare governance.’ This paper responds directly to this challenge, arguing that the underlying driver of payday lending is the confluence of three major trends that form part of the neo-liberal project: growing income insecurity for people both in and out of work; reductions in state welfare provision; and increasing financialisation. The state's response to payday lending in the UK has been regulatory reform which has effectively ‘regularised’ the use of high-cost credit (Aitken, 2010). This echoes the experience of Canada and the US where:

recent regulatory initiatives. . . attempt to resettle – and perform – the boundary between the economic and the non-economic by. . . settling its status as a legally permissable and legitimate credit practice (Aitken, 2010: 82)

At the same time as increasing its regulatory role, the state has withdrawn even further from its role as welfare provider. As we shall see, people are left to navigate the ever more complex mixed economy of welfare and mixed economy of credit in an increasingly financialised world.

## The neo-liberal project: labour market insecurity; welfare cuts; and financialisation

The UK has witnessed a series of fundamental, inter-related, long-term changes in the labour market, welfare reform and financialisation over the last 40 or so years as part of a broader neo-liberal project (Harvey, 2005; Peck, 2010; Crouch, 2011). These changes have combined to produce a highly favourable climate for the increase in payday lending and other forms of HCSTC or ‘fringe finance’ (also known as ‘alternative’ finance or ‘subprime’ borrowing) (Aitken, 2010).

The early seeds of these fundamental changes in the labour market can be traced to the 1980s, when employment legislation formalised the weakening of the trade unions and the growth of greater ‘flexibility’ in the labour market (Resolution Foundation, 2013a). This, alongside other socio-economic changes, produced growing wage inequality and job insecurity. Incomes have fluctuated since then and the picture is complex but the main trend has been for incomes in the middle to stagnate and those at the bottom to fall, producing the so-called ‘squeezed middle’ and ‘crushed bottom’ (Corlett and Whittaker, 2014; MacInnes *et al.*, 2014). The global financial crisis, from 2007–8 onwards, exacerbated these trends with an increase in unemployment from just over 1.5 million at the beginning of 2007 to a peak of nearly 2.7 million in 2011 (Rowlingson and McKay, 2014). While unemployment has more recently started to fall, jobs are no guarantee of avoiding poverty or financial insecurity. More than three million workers were ‘underemployed’ in 2013 (in other words, looking for additional hours of work). And there were around 1.4 million people with ‘zero hours contracts’ in 2014 (Rowlingson and McKay, 2014). Figures have recently shown, for the first time, that the majority of people living in poverty are in households where at least one adult has paid work (MacInnes *et al.*, 2014).

Clearly, those in low-paid, insecure work have faced major challenges to make ends meet (Resolution Foundation, 2013b) but those out of work face an even greater struggle. A detailed analysis of social security reforms over the last 40 years is well beyond the scope of this paper (see McKay and Rowlingson, 1999; 2008; forthcoming) but it is clear that the state has progressively withdrawn from providing adequate levels of support with a shift from a ‘redistributive’ and ‘provider’ welfare state to one based more on ‘regulation’, ‘investment’ and ‘activation’ (Klein and Millar, 1995; Morel *et al.*, 2011). As a result of various cuts, by 2015, means-tested benefits fell far short of a minimum income standard (MIS). A single person, out of work, was £100 short, per week, of reaching MIS in 2008, and £110 short in 2015. A lone parent with one child was £74 short, per week, of reaching MIS in 2008, and £118 short in 2015 (Hirsch, 2015).

One particular area of the social security system, the Social Fund, is highly relevant here. For decades, the Social Fund provided people on the lowest incomes with no-interest loans in times of need. The Fund was continually cut back until it was finally abolished by the Coalition government (2010–15) who transferred funding to local authorities in England to support the creation of local welfare schemes. This, however, led to a 75 per cent fall in provision in 2013–14 at a time when need was increasing (Gibbons, 2015).

Changes in the labour market and welfare state are also occurring alongside increasing financialisation on both a macro level (the increasing role of the finance sector in the UK economy) and a micro level (the increasing role of financial products in people's lives) (Langley, 2008; Heyes *et al.*, 2012; Clasen and Koslowski, 2013). Van der Zwan (2014) has identified three broad approaches to financialisation in the extensive literature on this subject. The first ‘regime of accumulation’ approach sees financialisation as a successor to the Fordist regime, providing a response to the decline of productivity from the late 1960s onwards by combining flexible labour markets with the expansion of finance/credit to maintain levels of consumption (Krippner, 2005 following Arrighi, 1994; see also Crouch, 2009). The precise link between these trends is contested, of course, with some seeing financialisation as the driver of labour market flexibility, for example, rather than as part of a broader neo-liberal ‘project’. We take the latter approach but nevertheless acknowledge these debates (see Dumenil and Levy, 2004; Kotz, 2010).

The second ‘shareholder value’ approach to financialisation focuses on the way that corporations have shifted their emphasis from investing profits (back) into the firm (not least through wages) to an emphasis on returning an increasing amount and proportion of profits to investors/shareholders. It would certainly be worthwhile to explore the role of the search for ever greater profits in the expansion of HCSTC but that is not the focus of this paper.

The third ‘financialisation of everyday life’ approach sees citizens being transformed from ‘welfare subjects’ to ‘personal investors’ and ‘personal borrowers’ with a related internalisation of new norms of individual risk-taking (Langley, 2008). Most accounts of the ‘everyday life’ of financialisation focus particularly on issues of culture, identities and subjectivities (Langley, 2008; Coppock, 2013; Deville, 2015; Horsley, 2015). This focus has provided a rich stream of thought about the nature of contemporary society but, we argue, fails to fully engage with the ‘lived experience’ or ‘lived reality’ of financialisation. Payday lending is not just important in terms of what it tells us about people's subjectivities and identities but also in terms of their more objective experiences of managing on low and precarious incomes. Van der Zwan (2014: 113–14) has also criticised the neo-Foucauldian emphasis on identities and subjectivities but from a different perspective, arguing that ‘the role of the state remains underdeveloped in this body of scholarly work. . . [and yet. . .] the expansion of financial markets has coincided with the retreat of the welfare state in many of the advanced political economies*’*. We also engage with, and contribute to, debates about the role of the state in this paper.

In bringing together the ‘regime of accumulation’ and ‘financialisation of everyday life’ approaches to our analysis of payday lending we also draw on discussion of the emergence of a ‘shadow’ welfare state (Fairbanks, 2009; Gottschalk, 2000). This relates to the varied sources of support people rely on from the mixed economy of credit (credit from different sources including the private sector, the state, family and friends and non-government microfinance schemes) alongside the mixed economy of welfare (Karger, 2005; Marston and Shevellar, 2014). In the US, for example, even before the global financial crisis took hold, the subprime lending industry paid out more money (by a factor of four to one) to poor families (in the form of loans) than was paid out by the state in the form of Temporary Assistance for Needy Families and the Earned Income Tax Credit combined (Committee on Ways and Means, 2008; Marston and Shevellar, 2014; Rivlin, 2011). While these trends may be particularly pronounced in the United States, the UK, has also experienced a major increase in HCSTC at a time of welfare state cuts.

Changes in the labour market, the welfare state and increasing financialisation are all clearly linked to each other and, as we have argued, can be seen as part of a more fundamental ‘neo-liberal project’, with its emphasis on de-(or re-)regulation, privatisation and individual responsibility (Aitken, 2010; Peck, 2010; Crouch, 2011). This transfer of risk and responsibility from the social/collective (welfare state) to the individual/personal (financial market) is clearly central to this project (Rowlingson, 2002; Finlayson, 2009). It is, therefore, no coincidence that payday lending has become most prominent in countries with highly financialised neo-liberal varieties of capitalism and liberal labour markets/welfare states such as the US and Australia, alongside the UK (Banks *et al.*, 2012; Gallmeyer and Roberts, 2009; Marston and Shevellar, 2014; Packman, 2014; Stoesz, 2012). This paper now provides an overview of the scale and nature of payday lending in the UK which has received remarkably little academic attention within social policy.

## The growth of payday lending in the UK

**Figure 1.**
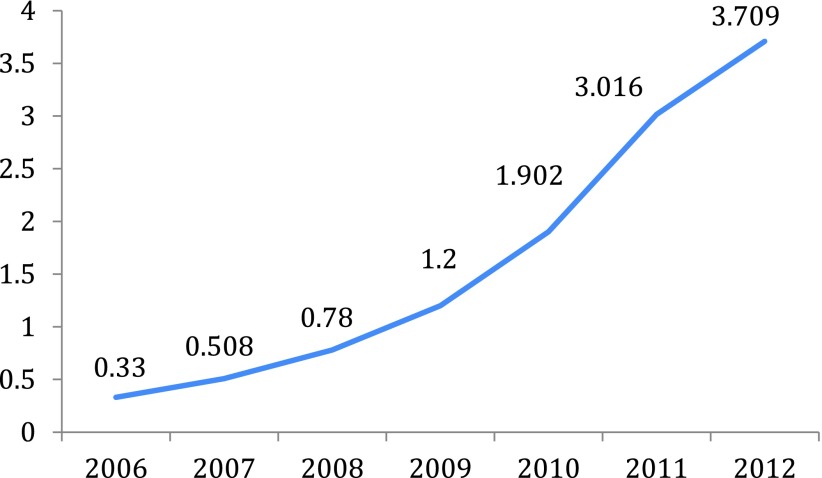
Amount of credit extended via payday loans (£ billions)

Source: Beddows and McAteer, 2014

Estimates of the size of the payday lending industry in the UK vary depending on definition and data source. Beddows and McAteer (2014) estimated that the amount of credit extended via payday loans had increased ten-fold from £0.33 billion in 2006 to £3.709 billion in 2012, with their definition including ‘traditional payday loans and short-term cash advances’ (Beddows and McAteer, 2014: 7) as shown in Figure 1.

The Competition and Markets Authority (CMA) (2014) estimated that, in 2012, there were 1.8 million payday loan customers in the UK, taking out approximately 10.2 million loans worth £2.8 billion. These figures are lower than the figures from Beddows and McAteer (2014) due to different definitions and data sources, but the CMA noted that their figures for 2012 represent a 35 to 50 per cent increase on the preceding financial year. So while precise figures vary, there is no doubt that payday lending grew phenomenally between 2006 and 2012.

The difficulties in agreeing on a precise definition of payday lending reflect the complexity of this market and its links to other forms of fringe finance (see also Rowlingson and McKay, 2014). Subprime borrowing has a long history in the UK with pawnbrokers and doorstep lenders being a prominent part of working class communities since the Victorian times, if not before (Tebbutt, 1983). But in recent years, the growth of payday lending has changed the face of this form of borrowing, quite literally from a very personal form to a more virtual one (though payday loans are also available through high street stores). This fast-changing evolution (in response to customer demand, investor appetite, technological change and new regulations) further complicates the nature of the industry. Technological developments have clearly facilitated the financialisation of everyday life (Davis, 2009). So, while payday lending itself is not new, the ability to access credit online within hours, if not minutes, would not be possible without modern credit scoring techniques and online platforms.

Concern about the rise of this form of lending^1^ led the Financial Conduct Authority and Competition and Markets Authority to carry out various (mainly quantitative) studies of the industry^2^ (CMA, 2014a; TNS/BMRB, 2014). They found that 60 per cent of payday loan customers were male and also young compared with the population as a whole. The median net income of an online payday lending customer was £16,500 in 2013 – broadly similar to that of the wider UK population (£17,500). However, 21 per cent of respondents said that they did not know what their household income was and so were not included in the median figure. And a further 23 per cent of customers stated that they had a variable income and so, again, were not included. Bearing in mind these data limitations, the distribution of payday loan customers’ incomes does seem somewhat narrower than that for the UK population – with fewer individuals on particularly low or particularly high incomes. This reflects, perhaps, the nature of the loan, which is intended for people in work but with low or irregular incomes. Indeed, more than eight in ten (83 per cent) payday lending customers were reported to be working (TNS/BMRB, 2014).

The CMA survey (CMA, 2014a; TNS/BMRB, 2014) also asked customers why they needed to take out a payday loan: 52 per cent of customers said that the loan was linked to an unexpected increase in expenses or outgoings; and 19 per cent said the need was due to an unexpected decrease in income. More than half (59 per cent) of customers said that they could not have gone without the item they purchased from the loan but, in a later question, 24 per cent of this group subsequently said that had payday loans not been available they would have gone without. Customers said that, when taking out the loan, they had been confident about their ability to repay it on time, but 17 per cent admitted that repaying the loan had been more difficult than they expected.

## Qualitative research with payday lending customers in the UK

The research from the CMA provides an important overview of the customer profile of payday lenders but it was never designed to explore the ‘lived experience’ or broader issues of interest within social policy, namely the links between labour markets, welfare state cuts and financialisation. In order to explore these issues in greater depth, we carried out AHRC-funded qualitative research (in-depth interviews) with 21 borrowers who had borrowed from payday lenders in the previous year. Fieldwork took place between March and June 2014 in the West Midlands and Oxfordshire regions of the UK. We recruited interviewees using a specialist company who identified people in shopping centres and high streets using a screening questionnaire the authors had designed. We interviewed a broad mix of participants in terms of age, gender, employment, family type and so on. Each interview lasted between 45 minutes and 2 hours at a place of the respondent's choice (the majority in their home, with some in a café). Where possible, the authors of the article conducted the interviews in pairs to ensure research quality and safety.

The research received full ethical approval by the University of Birmingham and we took ethical concerns seriously. We gained informed consent by explaining, at the beginning of each interview, the nature of our research, how the data would be used and this was also explained in our research information sheet which we gave to each participant. To thank the participants for their time (and encourage participation), we gave them £30 cash. This payment was initially queried by our university ethics reviewers and we appreciate the debate about paying respondents (Thompson, 1999) but we wanted to recognise the time and help that interviewees gave us. We also provided them with an information sheet with details of organisations providing free, confidential and independent advice on money issues, should this be of use. The interviews were carried out by the authors who are fully trained and experienced in carrying out interviews into potentially sensitive issues. We have used pseudonyms and other measures to ensure participant confidentiality.

Each interview was digitally recorded and transcribed in full. We scrutinised our data using thematic ‘framework’ analysis (Ritchie *et al.*, 2013) aided by Nvivo computer software. We had identified key themes from the literature and had a broad theoretical framework but remained open to new themes emerging from the data. The next part of this paper illustrates these themes. Our analysis clearly shows the role of poverty and precarity in causing a need for payday lending. We also show that people's views of payday lending were complex, with many positive aspects highlighted alongside the more familiar concerns about the great expense of this form of credit.

### Poverty and precarity

Respondents mentioned a range of reasons for needing to borrow money including: variable wages; insecure work (such as zero-hours contracts); self-employment; loss of employment; low levels of benefit income; loss of benefit income due to cuts and sanctions; and benefit delays. The following case studies are chosen as typical examples to illustrate this.

Amy was in her mid-20s living with her partner, Howard, and was the mother of a young child. Howard was paid on a weekly basis and their level of income would change from one month to another, depending on how much he earned from additional jobs and overtime. Amy had taken out a wide variety of credit products. She needed the credit for a range of things, including baby items and a replacement tumble dryer. She also, at one time, needed a loan to pay her rent due to delays with housing benefit:

I was on housing benefits at the time and my landlady didn't want to wait for the claim to go in and we were getting harassed and I was pregnant. I wasn't very well and basically, just to get peace and quiet, I went and got a [loan] out.

Sarah was 26 and a single mum with two children who had recently started working in hospitality on a zero-hours contract. She was already using a wide range of credit products, including home-collected credit, pawnbroking, payday loans and credit unions. Within the last twelve months, Sarah had borrowed from one online payday lender and one high-street payday lender even though she was unemployed at that time. Sarah used her loans, which totalled £440, ‘just to get by’, for her small children and for essentials such as *‘*food and electric and gas’.

Kate was a 28-year-old student living with her partner, who was self-employed. They saw payday loans as performing the same role as tax credits, basically a ‘top-up with the wages’.

Trixie was a 35-year-old single mum with two children, one of whom had recently turned 18. She lost her job in 2012 and turned to a small payday lender to help tide the family over until she was able to find new employment. She believed that it would be easy to obtain similar employment and therefore only took out £200 to help ‘bridge the gap’ and buy groceries and petrol for her car.

Georgina was an unemployed 19-year-old woman who lived with her mother (who was also out of work). She had borrowed from a number of online and high-street payday lenders. She commented that she originally needed the money:

just to help you through, you know, like, your Job Centre money and, you know, food and, I suppose you want to get bits of clothing for yourself, and you couldn't afford that most of the time, on that sort of money, because you've got to make it last you one week, and then the next week. And. . . sometimes you have to borrow before you get to your next payday, and then you have to pay them back, so it was just hard to survive.

### Positive aspects of payday lending

Contrary to the almost wholly negative portrayal of payday lending as ‘extortionate’ and ‘predatory’ by the media and campaigners, borrowers mentioned positive aspects of this form of credit in terms of the ease of access, and the ability to maintain dignity, privacy (especially in relation to online payday borrowing), responsibility and independence. And while the problematic aspects of payday lending certainly deserve attention (see next section), the majority of payday loans are, indeed, repaid on time (CMA, 2014a).

The borrowers in our sample generally appreciated the fact that the online application process for a payday loan was simple and quick. They liked the fact that they had access to credit the same day if not within an hour of their application being accepted. Some also liked the anonymity of the online process as they felt embarrassed or ashamed that they needed credit and did not want to feel judged. The desire to maintain dignity/avoid shame has not been previously highlighted in relation to payday lending but fits with recent research on the role of shame in relation to poverty (Walker, 2014).

Shame-avoidance was not the only reason for preferring online methods. In a few instances where people had used a face-to-face retail payday lender they said they had been treated unfairly or had even been offered *more* credit than they wanted.

Borrowers also generally saw borrowing as a means of managing their situation independently and responsibly. They were keen to avoid becoming a ‘burden’ on family and/or friends. However, in some cases, borrowers did eventually ask their family for help if they had suffered financial difficulties as a result of taking out the payday loan. And sometimes families intervened as soon as they became aware that a family member had used a payday loan.

Quick access to credit is a well-known feature of payday loans which is much debated, with some people arguing that access might be ‘too quick’ (CMA, 2014b; IPPR, 2014; Appleyard *et al.*, 2015). Less attention, however, is given to another feature that borrowers also generally valued – the fact that a payday loan could be *repaid* quickly. Many of the borrowers we interviewed were debt averse and liked the fact that the payday loan could be repaid quickly so that they would not have a debt hanging over them. The nature of payday loans is therefore very different from ongoing credit card debt or longer-term personal loans.

Olivia, for example, was 29, with a mortgage and a single parent to three young children. She was not currently working. Olivia took out a payday loan for £200 for Christmas presents as she explains:

it was around Christmas time, and I took out a payday loan, and I know I shouldn't, because I wasn't working, but they don't check, and I knew that I could pay it back. I could pay it back out of my tax credits, which wasn't a problem.

Olivia talked about the convenience of the application process:

If I remember rightly, I applied for the loan in the morning, and within, I think it was about 40 minutes, the money was there in my bank, and then I went out and spent it, as you do [laughs]. Then when it was time to pay it back, I got an email the day before, just reminding me that it was due to be taken out of my bank the next day, and it went really well. And then, obviously, when they've took the payment, they send you another email saying, ‘Thanks for the repayment, and come back at any time.’ So I just think it was so simple and easy. I didn't even have to speak to anybody, which was great.

Olivia liked the anonymity of the online process of payday loans:

I'd rather no one know, than go into a shop. I mean, you can walk into a shop and the person behind the counter, you might know them, and I'd rather, you know, just do it where no one knows; they don't know who you are.

In terms of the cost of credit, Ian thought that, on balance, the cost of the loan was proportionate to the ease and speed of the transaction:

I thought it seemed reasonable if I could get hold of it quickly, a hundred pounds isn't much to worry about. So with that in mind, I was like, that is reasonable. I know what I'm buying into. There wasn't anything hidden underneath any of that.

Some people also felt that payday loans allowed them to maintain their financial independence and dignity, and people were prepared to pay quite dearly for this. For example, Wayne was 38, working full-time and a father of two who was separated from his partner. Wayne borrowed £300 from an online payday lender to bridge a shortfall in his income. Wayne had considered other options first:

I did think about asking friends, family, you know . . . I suppose borrowing from friends and family you wouldn't have to pay the interest, or not some people anyway. But I think it was just an easier option for me, because I'm one for not asking people for anything; I'd rather try and do it myself.

Over the last five years, John had a number of loans from different payday lenders for car repairs and household bills when he was in insecure employment. If John was unable to access a payday loan he could have asked family to help. However, John preferred to handle the situation himself:

I didn't want to bother them [my family] by asking for the money, because obviously they'll start asking questions and worrying. I'm better off just sorting it myself.

John had also used home-collected credit once before but did not like the protracted repayment period or the agent calling at home. John preferred the quick ‘bullet’ one month repayment requirement because ‘I'd rather just get it out of the way’. John thought that this would be better because:

at least it's not on my mind then. I do worry a bit and I'm like, ‘Oh, I've got that [doorstep lender] to pay again this week’.

### A necessary evil?

While some aspects of payday lending were viewed positively, borrowers, in our sample, were certainly conscious that this was a very expensive form of credit but they believed that alternatives were either similarly expensive or, if cheaper, unsuitable or unavailable to them. Some alternatives, like credit union loans, may have been available, but awareness of credit unions was low.

As an example, at the end of each month, Amy and Howard sat down and worked out the family finances and whether they had enough funds to cover all their expenses. If not, they then checked to see if it would be cheaper to get a short-term payday loan to keep them going until Howard was paid next or whether it would be better to incur the bank charges associated with going into an overdraft. They found that payday lending was often cheaper than going into an overdraft situation. On one occasion they had used one payday loan for part of the deposit/first month's rent for a flat. When asked what she would have done if she did not have access to payday lending for this expense, Amy commented:

Do you know what? Even now I don't think I could have done it any other way. There was no-one else who could financially help because we exhausted all resources asking people . . . we didn't have enough because [money obtained from other sources] was the majority of the deposit and we had to find the first month's rent.

Amy was also still paying most of these loans off, and it appeared that she would be doing so for quite a while. She also had one payday loan that was ‘sitting at the bottom of a drawer’ in her house as she was ‘too scared’ to find out how much was left owing on the loan and did not want to tell Howard about this.

Olivia was aware that she was paying dearly for access to credit but this was not a significant enough issue to stop her from taking out another payday loan in the future:

I do think it's a bit extortionate, but I would still go back and have another one if I needed one. I wouldn't think twice. I suppose, when you need money, at the time, you don't really care how much you've gotta pay back do you, and you just take it.

Because of his previous experience with bankruptcy, Edward was reluctant to get any credit but, due to his urgent need to pay his electricity bill, believed that his payday loan could not have been avoided. When asked why he did not call the electricity company to try and organise a repayment plan, Edward stated:

I think it's worth paying twenty odd quid interest rather than getting twenty quid's worth of phone calls trying to do that, and then they probably put you on a higher tariff or they said that you've got to have. . . a prepay meter and you'd have more aggro that way.

He managed to repay the loan within the two-week period, as he did not want to get caught in a constant need for credit, stating:

because that's when they really start making money out of you. I think there's a charge for not paying it back and then they roll it over and they compound the interest up and that's how, you know, you see these people who are paying, who get these huge bills, it's just because they kept them rolling over and the compounded interest has gone up and up.

His comments highlight a keen insight into the business models of many payday companies and closely reflect the research completed by Beddows and McAteer (2014) on this issue and the statistics from the OFT (2013) which show that firms obtained approximately half of their profits from the 19 per cent of people who were unable to repay their loans.

Ian secured a payday loan to act as a ‘quick fix’ to meet the shortfall in his rental deposit to secure rented accommodation. He saw a payday loan as a last resort and would only access such loans in ‘extenuating circumstances’ in the future.

John explicitly stated that he had used payday lenders as a ‘safety net’ as there was no (or perceived to be no) alternative options. John had tried to access credit from his bank, however, as he explained:

I've got a bit of a bad credit. I did try with my bank, but as much as recently I've had good credit [but] they still look at your history status beyond that. I did look at that option, even getting an overdraft, but they just declined me. I felt as if I was pushed into a bit of a corner. I did say, ‘What else can I do?’

This case study reflects the limited alternative credit options people have if they do not have a good credit history. However, lack of awareness of cheaper options was also an issue. People were aware of payday loans through extensive advertising (in 2011, Wonga alone spent £16 million on advertising; Gentleman, 2013), as well as through recommendations from family and/or friends. More affordable forms of finance (such as credit unions) are rarely advertised, probably due to lack of funds but also due to concerns about ‘encouraging’ people to borrow through advertising, and concern that any increased demand would be difficult to meet.

As mentioned above, Trixie had used payday lending as a ‘bridging fund’ when her job came to an end but it took significantly longer than anticipated to find a new job and so her £200 loan quickly spiralled out of control until she owed £860. Even when she found employment, it was still difficult to repay the debt. As Trixie stated:

it got to a point where when I started my work. . . where every month. . . when I got paid I had about £2 to £3 left in my bank account so then I would have to re-borrow to get it back up so it was escalating, it was horrible, really bad. But I couldn't go to my bank, I couldn't get a low APR, I couldn't get lower repayments or anything.

Because of her escalating financial problems, Trixie used a large number of payday lenders (up to five at the same time) and had multiple loans to keep her going because of the shortfall left due to the repayments of previous loans.

## Discussion and policy implications

Drawing on an analysis of the ‘regime of accumulation’ and the ‘financialisation of everyday life’ perspectives, this paper demonstrates a clear and fundamental link between payday lending and changes in the labour market, welfare state and financialisation. Our new and rigorous qualitative interviews have shown how payday lending is the result of income insecurity and low incomes both in and out of work as people increasingly have little alternative but to borrow from high-cost lenders to try to make ends meet. Sometimes this leads to debt spirals and so compounds the problems they face. But in other cases, payday lending plays a positive role in bridging gaps in income. Technological advances in terms of quick credit scoring and online platforms are also important here and highly valued by many customers, not least for preserving anonymity and therefore dignity.

Our paper also makes a very particular contribution to academic debates about the ‘financialisation of everyday life’. Previous studies in this field (Langley, 2008; Coppock, 2013; Deville, 2015; Horsley, 2015) have focused on broad aspects of consumer credit and debt cultures from the perspective of changing subjectivities and identities. Our focus on the ‘lived experience’ of payday lending contributes to this alternative and more sophisticated picture of the role of payday lending in people's lives. The focus on ‘lived reality’ is important, in itself as a contribution to knowledge, but even more so because it facilitates a challenge to the dominant, though highly influential, portrayal of payday lending.

Indeed, this dominant portrayal of payday lending led the FCA to tighten regulation of HCSTC including new regulations from April 2014 (see FCA, 2014a for full details and Gardner, 2013 for discussion) with the result that the number of loans and the amount borrowed from payday lenders dropped by 35 per cent in the five months following the changes (FCA, 2014b). Many campaigners, however, argued for further regulation including a cap on the cost of credit. The FCA therefore consulted on this and estimated in November 2014, that 7 per cent of current borrowers – some 70,000 people – may not have access to payday loans following the introduction of their proposed price cap (FCA, 2014b). They further claimed that these people would be better off without access to payday lending. We submitted evidence from our research to the FCA in 2014, arguing that, for some people, the proposed price cap was likely to have a more harmful than positive effect unless alternatives were put into place (Rowlingson *et al.*, 2014). This was for a number of reasons. First, home-collected credit was excluded from the cap, so some people might seek credit from this similarly expensive source despite the lack of anonymity and other features which our research showed people valued. People might also make use of overdraft facilities which our research also highlighted can be more expensive than payday lending (as they, again, are not subject to a price cap). And while credit unions are currently being funded to modernise and expand, they still lack the capacity to provide the scale of loans, with the likely level of default that would be needed. Illegal lending may also increase as a result of these reforms though this is hotly debated (PFRC/Policis, 2006; Gibbons, 2012).

We are not seeking to deny, in this paper, that payday lending is an extremely expensive form of credit which can lead people into highly problematic debt situations. We do, however, argue that a more critical analysis of the root causes of the growth of payday lending, along with a better understanding of the ‘lived reality’ of payday borrowing provides an important basis for a robust analysis of policy options. We have shown that the regula(risa)tion of payday lending will do nothing to tackle the root causes of demand for this form of credit which looks set to increase as recent welfare reforms, including various benefit caps and tax credit cuts, will hit the poorest ever harder (IFS, 2013; Beatty and Fothergill, 2013; Hood and Phillips, 2015; Lupton *con al*., 2015). The shift in the nature of the state from provider to regulator looks set to become further entrenched. And while there are some signs that employment and wages are increasing, much more needs to be done to improve job security and levels of pay, for example through substantial increases in the National Minimum Wage.

Nor are we seeking to deny, in this paper, that recent reforms, including the price cap introduced in January 2015, are likely to benefit more people than they will harm; but we are suggesting that some people will be worse off unless alternatives are put in place. These alternatives could include an expansion, and reform, of local welfare assistance to provide interest-free (or low-interest) credit alongside further support for credit unions. And (some of) this could be funded by the mainstream banks as with the Good Shepherd schemes in Australia^3^. The high cost of overdrafts, credit cards, rent-to-buy, logbook loans and doorstep lending also needs more attention as these have not been captured by recent reforms. Various other changes would also be helpful including: reducing benefit delays; providing more money/debt advice; and ensuring that utility companies effectively support people who struggle to pay bills. But, our over-arching point is that it is only through theoretically-informed and empirically-rigorous research that we can identify appropriate policy responses to payday lending within the context of the broader mixed economies of welfare and credit. We hope this paper makes a useful contribution here.

## Conclusion

Personal finance issues have not been widely explored by social policy academics and yet, as argued here, they go to the heart of the changing nature of the state and the mixed economy of welfare/credit. The problem of payday lending lies in the deep roots of neo-liberalism as manifest through labour market insecurity, welfare cuts and financialisation. Calls for reform of payday lending have generally ignored this broader perspective and have, instead, been based on a relatively superficial and wholly negative, though extremely influential, account of payday lending. Our rigorous empirical research on the ‘lived reality’ of payday lending provides a more sophisticated and balanced picture. We have argued that, while all else remains equal, it is clearly important to regulate this, and other forms of, credit appropriately but such regula(risa)tion acts to normalise this form of credit and can also have unintended, negative, consequences for some. It is therefore important for social policy academics, campaigners and policy-makers to engage more with theoretically-informed and empirically-rigorous research on personal finance issues and, in the specific case of payday lending, to understand this within the context of the broader neo-liberal project and the lived reality of the ‘mixed economy of credit’ and ‘shadow welfare state’.

## Notes

1 In 2013, payday loan companies reported that the average cost of borrowing £100 was around £25, but ranged from £14 to £51 (OFT, 2013). To give a particular example, Wonga, in 2014, lent £150 over 18 days and charged interest of £27.99. The transmission fee was £5.50 so the total cost would equate to a representative 5,853% Annual Percentage Rate (APR).

2 The CMA/TNS/BMRB research included a telephone survey of 1,560 payday lending customers carried out in 2013 and transaction data on 15m loans from 11 of the largest providers.

3 See details of various loans and savings schemes at http://goodshepherdmicrofinance.org.au/which-loan-right-me
